# Bordetella bronchiseptica pneumonia in a patient with lung cancer; a case report of a rare infection

**DOI:** 10.1186/s12879-017-2736-7

**Published:** 2017-09-25

**Authors:** Manlio Monti, Danila Diano, Francesco Allegrini, Angelo Delmonte, Valentina Fausti, Paola Cravero, Giulia Marcantognini, Giovanni Luca Frassineti

**Affiliations:** 1 Department of Medical Oncology, Istituto Scientifico Romagnolo per lo Studio e la Cura dei Tumori (IRST) IRCCS, Meldola, Italy; 2 Radiology Unit, Istituto Scientifico Romagnolo per lo Studio e la Cura dei Tumori (IRST) IRCCS, Meldola, Italy; 3 Department of Infectious Diseases, Morgagni-Pierantoni Hospital, Forlì, Italy

**Keywords:** *Bordetella bronchiseptica*, Cavitary lung lesion, Rare infection, Nivolumab, Nosocomial infection

## Abstract

**Background:**

*Bordetella bronchiseptica* (*B.bronchiseptica*) is a frequent cause of respiratory infections in animals but rarely causes serious infection in humans. We present a rare case of *B. bronchiseptica* pneumonia in a patient with lung cancer.

**Case presentation:**

A 52-year-old white male with non small cell lung cancer developed fever during treatment with nivolumab. A persistent productive cough and a deterioration in his clinical condition led to his hospitalization for evaluation. Bronchoscopy was performed and a diagnosis of *B. bronchiseptica* pneumonia was made. The infection was successfully managed by antiobiotic therapy.

**Conclusions:**

*B. bronchiseptica* is a pathogen that can cause serious infection in humans, especially in immunocompromised or immunoincompetent individuals. In our patient it showed unusual resistance to cephalosporins and poor sensitivity to amikacin. To our knowledge this is the first case of such an infection in a lung cancer patient undergoing treatment with nivolumab. When B*. bronchiseptica* is identified, the possibility of a nosocomial transmission must be considered.

## Background

Of the 9 species of *Bordetella* (*B.*) identified to date, *B. bronchiseptica*, *B. parapertussis* and *B. holmesii* are associated with respiratory infections in humans and other mammals. Whooping cough (pertussis) is a highly contagious, acute respiratory illness of humans caused by the gram-negative bacterial pathogen *B. pertussis, which has* no known animal or environmental reservoir [1]. *B. avium* is a bird pathogen that causes coryza and rhinotracheitis in poultry. *B. ansorpii*, *B. hinzii*, *B. holmesii*, *B. petrii* and *B. trematum* are mainly associated with infections in immunocompromised patients [2, 3].

*B. bronchiseptica* is a gram-negative coccobacillus frequently isolated in the upper respiratory tract of domestic animals and wild animals, e.g. voles, seals, rodents and captive koalas. The infection is believed to be transmitted from animals with tracheobronchitis. Despite potentially frequent exposure to zoonotic sources of this opportunistic pathogen, human infections are rare. However, it is known to be an etiologic agent of upper respiratory tract infections, pneumonitis, endocarditis, peritonitis, meningitis, sepsis and recurrent bacteremia in immunocompromised or immunoincompetent patients [4]. *B. bronchiseptica* does not express pertussis toxin, which is one of the characteristic virulence factors of *B. pertussis*, but has the genes to do so, indicating the close evolutionary relationship between the two species. We report a rare case of *B.bronchiseptica* pneumonia in a patient with lung cancer.

## Case presentation

**Fig. 1:**
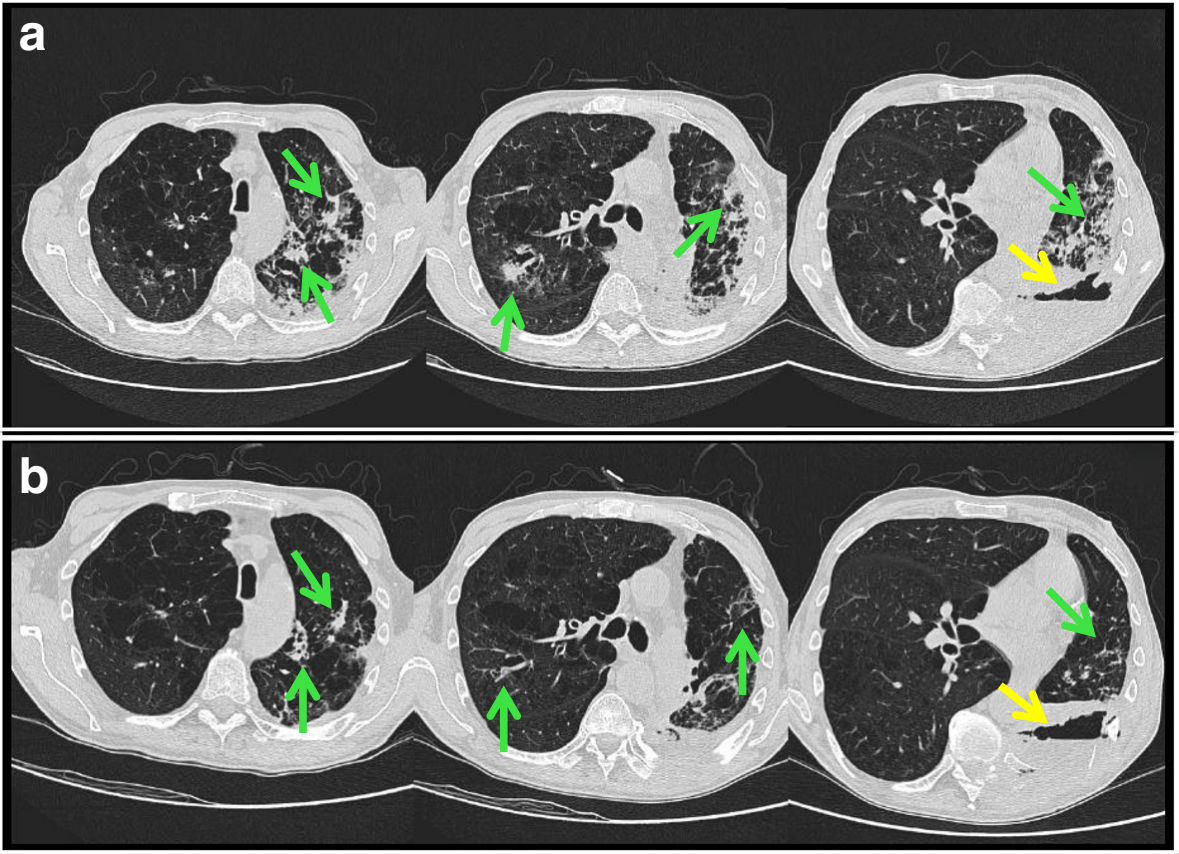
Axial chest CT scans during hospitalization. Pre-treatment images (**a**) show the cavitary lesion of primary lung cancer (yellow arrow) and multiple bilateral pulmonary consolidations, ground glass opacities and septal thickening prevalent in the left lower lobe relating to inflammatory phenomena (green arrows). Images after 2 weeks of piperacillin/tazobactam therapy (**b**) reveal a stabilization of the cavitary lesion (yellow arrow) and a reduction in the inflammatory phenomena (green arrows)

In April 2014, a 52-year-old Caucasian male with a history of cigarette smoking was diagnosed with cavitating squamous cell lung carcinoma (SCC) cT4N2M0 of the inferior left lung lobe. Between August 2015 and January 2016, he underwent 10 cycles of second-line treatment with nivolumab (3 mg/kg every 14 days) at our institute. Fourteen days after the end of the last treatment the patient developed low-grade fever (38.3 °C). A chest X ray excluded the hypothesis of pneumonia. Levofloxacin 500 mg/day was prescribed for 7 days and the patient initially responded. Two further cycles of nivolumab were administered. At a follow-up appointment in February 2016, the patient reported having had low-grade fever (38 °C) for about a month and complained of pain in his chest wall over the area of the lung cancer. He also had a productive cough and had lost around 7 kg over a period of 6 months. Performance status was poor. The patient was urgently admitted to our inpatient oncology ward; initial vital signs were blood pressure 110/60, heart rate125 beats/min and oxygen saturation 92% in room air. Auscultation of the patient’s lungs revealed decreased breath sounds over the whole lung field. The abdomen was soft, without organomegaly. Laboratory exams showed a white blood cell count of 22.10 × 10^9^/L with 84.5% neutrophils, 4.7% lymphocytes and 10.5% monocytes. Hemoglobin was 10 g/dl and platelets 690 × 10^9^/L. Electrolytes, renal and liver function tests and coagulation studies were all normal apart from C-reactive protein (320 mg/L) and albumin (28 g/L). Urine analysis showed protein 25 mg/dl. A chest X ray confirmed the presence of the cavitary lesion of the primary tumor and pleural effusion. A blood culture was performed and empirical treatment was started with amikacin 20 mg/kg on day 1 (15 mg/kg/day thereafter) plus ceftazidime 2 g 3 times/day. The patient also started oxygen therapy (oxygen therapy eyeglasses) 5 L/min and became apyretic after 3 days of the dual antibiotic treatment. Bronchoscopy was performed to investigate the continuous productive cough and foul-smelling pus was removed, mainly from the left lower lobe. The patient’s clinical conditions worsened after 7 days of the dual therapy, with a reappearance of fever and chills. A chest computerized tomography (CT) scan revealed the presence of multiple bilateral parenchymal accumulations mainly in the left lower lobe, leading to a first hypothesis of inflammatory phenomena (Fig. 1a). Minimal left pleural effusion was also noted, together with an increase in the size of the cavitary lesion in the inferior left lobe and increased lytic rib disease in the adjacent vertebral structures. Treatment with amikacin was maintained but ceftazidime was replaced by teicoplanin 6 mg/kg twice on the first day and once a day thereafter. Blood culture remained negative. Tests for human immunodeficiency virus (HIV) and quantitative cytomegalovirus DNA (< 233 UI/ml) were negative. Bronchoscopic alveolar lavage (BAL) revealed the presence of *B. bronchiseptica* 10,000 CFU (colon-forming units) which was resistant to cefepime (minimal inhibitory concentration [MIC] ≥ 64), cefotaxime (MIC ≥64) and ceftazidime (MIC 16), but sensitive to amikacin (MIC 8), gentamicin (MIC 2), imipenem (MIC 0.5), meropenem (MIC ≤0.25), piperacillin/tazobactam (MIC ≤4) and ciprofloxacin (MIC 1). The bronchoalveolar material was negative for *Mycobacterium tuberculosis* complex DNA. After several days of apyrexia the fever returned and the patient’s clinical conditions deteriorated. A blood culture was repeated and amikacin and teicolplanin were replaced by piperacillin 4 g plus tazobactam 500 mg three times/day. After 2 weeks’ therapy, the chest CT scan (Fig. 1b) showed an improvement in the bilateral parenchymal inflammatory areas and a slight reduction in the pleural effusion. Blood culture remained negative. Antimicrobial treatment was continued for a total of 17 days after which, given the improvement in the pneumonia, clinical conditions and performance status, the patient was discharged. Levofloxacin 500 mg twice a day was prescribed for the first 6 days at home. The patient died 3 months later due to disease progression.

## Discussion

Of the nine species of *Bordetella, B. bronchiseptica* is primarily a zoonotic pathogen associated with a wide spectrum of illnesses ranging in severity from mild respiratory symptoms to sepsis and death in humans [5]. In a review of the literature, the majority of patients infected with *B. bronchiseptica* had at least one predisposing disease such as acute lymphatic leukemia [6], chronic lymphocytic leukemia, lymphopenia associated with temozolomide treatment for glioblastoma, cystic fibrosis [7] and cancer (malignant thymoma, brain metastases of unknown origin, lung cancer and supraglottic cancer with no evidence of recurrence) [8], or had undergone hematopietic stem cell transplantation [9–11] or a lung transplant [12]. *B. bronchiseptica* has been reported in individuals with HIV [13], Down’s syndrome, branchial cleft cyst [14], Crohn’s disease, thoracic trauma, endocarditis, diabetes with “healed” pulmonary tuberculosis, post-traumatic meningitis, rheumatoid arthritis [15], and bronchitis [16].

Although little is known about the route of transmission of *B. bronchiseptica* infection, airborne human to human transmission has been reported in hospital settings [8, 10]. Our patient thus required droplet precautions and was placed in a single room.

*B. bronchiseptica* infects a wide range of mammals, causing tracheobronchitis (also known as ‘kennel cough’) in dogs and cats and atrophic rhinitis in pigs. Kennel cough vaccines containing live, attenuated *B. bronchiseptica* are commonly used in veterinary clinics. Transmission of vaccine strains to humans is a theoretical possibility [17]. When infections due to *B. bronchiseptica* occur in humans, they typically involve immunocompromised or immunoincompetent patients and are often acquired through contact with animals. The pathogen can survive intracellularly for several days after uptake by phagocytes, which may, in part, explain the persistence of infection in immunoincompetent individuals. Our patient had been a frequent visitor to a cat shelter a few years previously and his infection may thus have originated from an animal.

Lung cancer is the leading cause of cancer death in the world. In 2008, more than 1.6 million people were diagnosed with the disease, representing 13% of all new cancer diagnoses, and 1.4 million died, representing 18% of all cancer deaths [18]. Lung cancer is divided into two major classes based on its biology, therapy and prognosis, i.e. non small cell lung cancer (NSCLC) and small cell lung cancer (SCLC). The former accounts for more than 80% of all lung cancer cases and includes 2 major subtypes: non-squamous carcinoma of the lung (including adenocarcinoma, large-cell carcinoma, and other types) and squamous cell carcinoma (SCC). SCC accounts for 20%–30% of all lung cancers, representing a significant health burden [19]. The last 5–10 years have seen substantial advances in the development of molecular targeted therapies for advanced-stage lung adenocarcinoma, improving the outlook for patients with this disease. However, until recently, treatment for patients with advanced SCC was limited [20] and based on histologic type and performance status. Median overall survival for stage III B SCC is currently around 18 months. Our patient showed a slightly better overall survival and the *Bordetella* infection probably did not modify prognosis [21].

Advances in our understanding of the interaction between the immune system and tumors have led to the development of programmed death-1 (PD-1)/programmed death ligand-1 (PD-L1) inhibitors targeting the immune checkpoint pathway. The monoclonal antibody nivolumab was the first immune checkpoint inhibitor approved by the U.S. Food and Drug Administration for the treatment of advanced SCC and NSCLC following progression during or after platinum-based chemotherapy. Nivolumab binds the receptor PD-1, expressed in tumor-infiltrating lymphocytes, with high affinity. Its ligand, PD-L1, is expressed in different cancer types, including NSCLC, and PD-1/PD-L1 binding results in the suppression of the immune response. Nivolumab acts as a competitive agent and a PD-L1 antagonist, permitting a correct functioning of the immune system. It is a well tolerated treatment. Pneumonia is a possible side-effect of immune checkpoint inhibitors and nivolumab-associated pneumonia has been observed in melanoma patients (around 2%) and in those with renal cancer or NSCLC (around 5%). However, the presence of any symptom requires the interruption of the drug and the initiation of steroid treatment. In the case of our patient, methylprednisolone 20 mg/day was started on the 5th day of hospitalization.

*B. bronchiseptica* rarely responds to macrolide antibiotics [1, 4] but is usually susceptible in vitro to aminoglycosides (amikacin, gentamicin), antipseudomonal penicillins (mezlociclin, piperacillin, ticarcillin), quinolones, tetracycline, third-generation cephalosporins (cefoperazone, cefatazidime) and trimethoprim-sulfamethoxazole [22]. Conversely, in our patient, *B. bronchiseptica* was resistant to cephalosporins. Of note, the clinical response to the above antibiotics is sometimes disappointing but the discrepancy between in vitro sensitivity and in vivo efficacy is not clearly understood. The first antibiotic we used was levofloxacin, a member of the fluoroquinolone family which is considered an effective treatment option for acquired pneumonia and acute exacerbations of chronic bronchitis [23]. We then tried amikacin plus ceftazidime as empirical antibiotic therapy because this combination has broad-spectrum antibacterial coverage and synergistic bactericidal activity [24]. When the infection did not resolve we administered teicoplanin as it covers gram-positive organisms, but then changed to piperacillin/tazobactam on the basis of BAL results. Treatment duration for *B. bronchiseptica* is a controversial issue and varies from 2 to 4 weeks for patients with a good response [14], and from 2 weeks to 6 months for patients with recurrent symptoms. Therapy with piperacillin/tazobactam was maintained for more 2 weeks in our patient. The route of infection in our patient remains unknown but was probably caused by an animal. He had no recurrence of fever but died 3 months after being discharged due to cancer progression.

Although there are no distinctive radiographic features associated with *B. bronchiseptica* pulmonary infection, the most frequent manifestations are multiple lobular infiltrations, interstitial pneumonia [13], and lobular pneumonia [6, 9, 13]. Cavitary lesions are rarely encountered [13, 25]. The case we describe is noteworthy for a number of reasons. First, *B. bronchiseptica* was resistant to ceftazidime (an unusual occurrence) and showed poor sensitivity to amikacin. Secondly, to our knowledge this is the first case of pneumonia caused by *B. bronchiseptica* in a lung cancer patient undergoing nivolumab treatment. The diagnosis of cavitary lung cancer was made in 2013, but by July 2015 the tumor had increased in size. Thus, we do not know whether *B. bronchiseptica* was present at the diagnosis of the lung cancer or whether the cavitary lesion increased the patient’s susceptibility to infection.

In conclusion, although *B. bronchiseptica* is a zoonotic pathogen, it can cause serious infection in humans, especially immunocompromised or immunoincompetent individuals. When *B. bronchiseptica* is identified, the possibility of nosocomial transmission must be taken into consideration. Lung cancer, bronchiectasis, emphysema, cystic fibrosis and other lung diseases causing structural changes may also be associated with *B. bronchiseptica* infection.

## Abbreviations

*B*: *Bordetella*
CFU: Colony-forming unit
CT: Computerized tomography
HIV: Human immunodeficiency virus
MIC: Minimal inhibitory concentration
